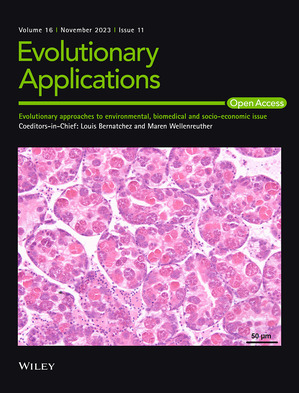# Cover Image

**DOI:** 10.1111/eva.13413

**Published:** 2023-11-27

## Abstract

Caption: Section of the digestive gland of a cockle infected with Marteilia cochillia.

Credit: Dr. Antonio Villalba.